# Performance of High Temperature Polymer Electrolyte Membrane Fuel Cells as a Function of Polybenzimidazole Membrane Modification

**DOI:** 10.1002/cssc.202501575

**Published:** 2025-10-21

**Authors:** Julia Müller‐Hülstede, Dana Schonvogel, Julian Büsselmann, Jörg Belack, Jurica Vidakovic, Md Raziun B. Mamtaz, Quentin Meyer, Chuan Zhao, Peter Wagner

**Affiliations:** ^1^ German Aerospace Center (DLR) Institute of Engineering Thermodynamics Carl‐von‐Ossietzky‐Str. 15 26129 Oldenburg Germany; ^2^ BASF Catalysts Germany GmbH Pettenkoferstrasse 9 67063 Ludwigshafen am Rhein Germany; ^3^ Trigona Fuel Cell Components GmbH Kasteler Straße 45 65203 Wiesbaden Germany; ^4^ School of Chemistry University of New South Wales Sydney NSW 2052 Australia

**Keywords:** fuel cells, high‐temperature polymer electrolyte membrane fuel cells, membrane‐electrode assembly, membranes, polybenzimidazole

## Abstract

Polymer electrolyte membranes (PEM) of high‐temperature PEM fuel cells (HT‐PEMFC) are commonly based on phosphoric acid‐doped polybenzimidazole (PBI). However, these membranes suffer from acid leaching and limited mechanical stability. In this study, five different PBI membrane modifications, including inorganic fillers (SiC, Si_3_N_4_, SiO_2_), crosslinking, and high‐solid content, are explored to increase the solid content of the membranes (up to 18 wt%), which could potentially increase mechanical stability. All modifications are based on the industrial fabrication process of the commercial Celtec‐P membrane to enable direct comparison in HT‐PEMFC. HT‐PEMFC testing reveal comparable performance to the Celtec standard when incorporating SiC particles with lower membrane resistance. Lowest performance is found for crosslinked and high‐solid‐based membrane electrode assembly (MEA), which is traced back to acid leaching and increased proton transport resistances. The evaluation of performance under operation with reformate reveal no beneficial effect of the membrane modification. This study helps to implement novel HT‐PEM candidates in the industrial fabrication process and provides direct comparison to the already commercialized Celtec technology regarding MEA performances and stabilities.

## Graphical abstract

This study helps to implement novel high‐temperature proton exchange membrane candidates in the industrial fabrication process and provides direct comparison to the already commercialized Celtec technology regarding membrane electrode assembly performances. Five different modifications toward higher solid content in membrane are tested, including inorganic filler and crosslinking.

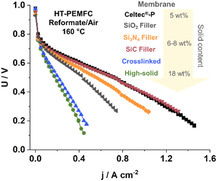

## 1 Introduction

Polymer electrolyte membrane (PEM) fuel cells are part of a renewable energy supply and provide energy conversion based on hydrogen. High temperature PEM fuel cells (HT–PEMFCs) are operated at an enhanced temperature of around 120–200 °C, which simplifies water and heat management and increases tolerances toward fuel contaminants (e.g., CO and H_2_S).^[^ ^1^, ^2^ ^]^ Thereby, HT‐PEMFCs are highly attractive toward the direct use of reformate from streams such as methanol or natural gas.^[^ ^2^ ^]^ However, HT‐PEMFCs still have many challenges to overcome for a longer lifetime and higher performance. With a look to the membrane, the commonly used phosphoric acid (PA)‐doped polybenzimidazole (PBI) membrane suffers from i) acid leaching, ii) reduced mechanical strength with increased PA doping and creep, iii) reduced proton conductivity with increased temperatures due to water evaporation and condensation of the PA after prolonged operations in HT‐PEMFCs.^[^ ^2^, ^3^ ^]^

Several membrane optimization strategies have been explored to increase the acid doping amount to enhance performance while maintaining stability and minimizing creeping by increasing the molecular weight of the polymer or the solid content. This includes ionic and covalent crosslinking and the addition of filler materials.^[^ ^2^, ^4^ ^]^ Özdemir et al. compared four different crosslinkers for PBI, namely diglycidyl ether (BADGE), ethylene glycol diglycidyl ether (EGDE), α‐α′‐dibromo‐p‐xylene (DBpX), and terephthalaldehyde (TPA).^[^ ^4^ ^]^ They found the best performance for the PBI/BADGE with a peak power density of 0.12 W cm^−2^ (H_2_/air, 2.5 bar), although 40% lower proton conductivity at 165 °C compared to PBI was found, but 14% higher acid retention.^[^ ^4^ ^]^

Regarding inorganic additives, SiO_2_,^[^ ^5^, ^6^ ^]^ imidazole‐functionalized silica,^[^ ^7^ ^]^ SiC,^[^ ^8^ ^]^ metal organic frameworks like ZIF‐8 or ZIF‐67,^[^ ^9^ ^]^ UiO‐66,^[^ ^10^ ^]^ carbon nanotubes,^[^ ^11^ ^]^ graphene oxide,^[^ ^12^ ^]^ or TiO_2_^[^ ^13^ ^]^ were used as fillers or crosslinkers. Incorporation of SiO_2_ particles can enhance the acid retention through their hygroscopic character.^[^ ^14^ ^]^ Devrim et al. showed that the introduction of SiO_2_ into PBI leads to a 13% higher proton conductivity at 165 °C and 13% lower acid leaching compared to PBI. Moreover, a slightly higher power density of 0.24 W cm^−2^ compared to PBI (0.20 W cm^−2^ at 165 °C (H_2_/air, 1.5/2.5) was reported. SiC particles are chemically stable under HT‐PEMFC conditions and have high strength and stiffness, which can enhance the mechanical stability of HT‐PEM.^[^ ^8^, ^15^ ^]^ Our previous study showed that the use of SiC as filler in PBI membrane with two different particle diameters (<1 and 2 μm) results in a higher power density at 1.0 A cm^−2^ (H_2_/O_2_) by up to 5% (657  and 646 mW cm^−2^) than commercial Celtec membrane electrode assembly (MEAs) (624  and 615 mW cm^−2^). Moreover, a lower cell degradation rate over a load‐cycling test between 0.6 and 1.0 A cm^−2^ for 1,000 h (−3 and 63 μV h^−1^ for SiC MEA vs. –101 and −243 μV h^−1^ for reference MEA at 0.6 A) was observed as well as lower ohmic resistance, better acid retention, and less membrane creeping.^[^ ^8^ ^]^ Similar to SiC, Si_3_N_4_ particles have comparable properties with lower wear resistance but higher flexural strength.^[^ ^16^ ^]^ However, their incorporation into HT‐PEM has not been reported so far.

There is a very large number of studies on PBI membrane modifications showing increased HT‐PEMFC performances for the modifications compared to a PBI reference.^[^ ^4^, ^6^ ^]^ However, most studies are based on home‐made PBI references with lower performances to state‐of‐the‐art HT‐PEM MEAs and show no comparison to commercial MEAs like Celtec and realistic operation conditions by using reformates. This study provides the HT‐PEM single cell operation of MEAs with differently modified polybenzimidazole (PBI) membranes based on Celtec and reveals success and failure in typical PBI modification strategies alike. This includes a crosslinked membrane, addition of the inorganic fillers SiO_2_, SiC, and Si_3_N_4,_ and an MEA with higher solid content by a factor of 4 compared to the standard Celtec membrane. High comparability of all tested MEAs is given by identical MEA fabrication and cell operation parameters to observe the PBI membrane effect. Along with cell operations using H_2_/O_2_, H_2_/air, and reformate/air, polarization curves, cyclic voltammetry (CV), and electrochemical impedance spectroscopy (EIS) give insights into MEA properties and behavior under HT–PEMFC conditions.

## 2 Experimental Section

### 2.1 Membrane

For the fabrication of Celtec‐P and novel membranes, the patented polyphosphoric acid (PPA) process by BASF Catalysts GmbH (Germany) was used.^[^ ^8^ ^]^ As starting material for the novel membranes, the PBI solution from Celtec‐P production was used.

### 2.2 Membranes with Inorganic Filler

As filler materials, SiC, Si_3_N_4,_ and SiO_2_ with mean particle diameters <10 μm were used. The synthesis for all filler membranes was analogous to our study published previously.^[^ ^8^ ^]^ A solution of polyphosphoric acid (PPA) with a PBI content of ≈2.5 wt% was held at 140 °C for 1 h, and phosphoric acid was added dropwise to adjust the viscosity. Then, 2 wt% of inorganic filler was added to the diluted solution, followed by heating to 160 °C and holding that temperature for 1 h. The resulting polymer suspension was cast onto a clean glass plate, and the obtained membrane was hydrolyzed in a bath with phosphoric acid (≈50 wt%) for 24 h.

### 2.3 High Solid Membrane

Isophthalic acid (IPA), terephthalic acid (TPA), and 3′,4,4′‐tetraaminobiphenyl (TAB) in a molar ratio of 0.125:0.875:1 were mixed under an inert atmosphere. 10 wt% of the monomer mixture was added to 666 mL of PPA under stirring in an inert gas atmosphere, followed by the addition of 333 mL of PPA and stirring for 1 h. Then, the mixture was heated in a temperature ramp up to 195 °C in 7 h. Afterward, the suspension was cooled down to 170 °C for 1 h with simultaneous addition of 120 mL phosphoric acid (25 wt%), followed by evacuation and ventilation with N_2_. The polymer solution was then cast onto a cleaned glass plate.

### 2.4 Crosslinked Membrane

Ortho phosphoric acid (85 wt%) and glutaraldehyde solution (50 wt% in water) were mixed to get a 1 wt% glutaraldehyde solution. The solution was heated up to 70 °C, and a common Celtec–P membrane was inserted for 1 h for crosslinking. Then, the membrane was put between two glass plates and put in an oven at 160 °C for 30 min, followed by insertion in 50 wt% PA at room temperature overnight.

### 2.5 Membrane Electrode Assembly

MEAs with an active area of 20.25 cm^2^ were prepared based on the Celtec‐P technology. The components are Celtec‐P or modified membranes and gas diffusion electrodes (GDE) on the cathode and anode side with woven gas diffusion layers with microporous layer with a total Pt loading of 1.8 mg_Pt_ cm^−2^.^[^ ^17^ ^]^ The cathode consists of platinum alloy, while the anode consists of platinum. All tested MEAs were fabricated similarly to maximize the comparability of experimental results and to make the effects of the modified membranes visible.

### 2.6 Test Procedure and Electrochemical Characterization

The test bench C1000 LT from HORIBA FuelCon GmbH (Germany) was used to operate the HT‐PEMFC single cells. For MEA tests, a cell fixture (cF25/100) graphite‐compound flow fields with a five‐fold serpentine structure and a compression unit from balticFuelCells GmbH (Germany) were used, equipped with CMD V1.1 unit from balticFuelCells. The test procedure was kept the same to ensure comparable experimental results. The cell compression was 0.75 MPa for the whole test duration without applying backpressure. The cell was heated up to 120 °C under a nitrogen supply on the cathode and anode. Then, the dry reaction gases (H_2_/air) were supplied with a stoichiometry of 1.5/2.0, and the electrical load was switched on simultaneously to slowly increase the applied current density up to 0.3 A cm^−2^. After that, the cell was further heated to 160 °C to start the break‐in for at least 48 h at constant load. To compare cell performances and behavior using different MEA types, an electrochemical characterization was carried out. Therefore, the test bench was used in combination with the potentiostat Modulab 2100 A from Solartron Analytical (UK). First, a polarization curve under H_2_/air (1.5/2.0) was recorded with a minimum voltage of 0.2 V and a maximum current density limit of 2 A cm^−2^. The step size was 0.5 A using a holding time of 30 s per step. In the next step, electrochemical impedance spectra (EIS) were recorded at current densities of 0.03, 0.1, 0.2, 0.3, and 0.4 A cm^−2^ with a frequency range of 100 kHz to 100 mHz. Afterward, a similar procedure was carried out under reaction gases of H_2_/O_2_ (1.5/9.5) and reformate/air (H_2_ 1.5/2.0), respectively. The reformate contains dry gases with 68% H_2_, 22% CO_2_, 9% N_2,_ and 1% CO. Last, the anode and cathode gas supply were switched to H_2_/N_2_ with constant flow rates of 0.1 L min^−1^, and the load was turned off. After 10 min, seven cyclic voltammograms were recorded between 0.05 – and 1.0 V_RHE_ with a scan rate of 100 mV s^−1^. The flow rates were increased to 0.3 L min^−1^, and linear sweep voltammetry was recorded between 0.19 and 0.50 V_RHE_ with a scan rate of 2 mV s^−1^. For electrochemical active area (ECSA) analysis, the hydrogen underpotential deposition (HUPD) with the signal of H‐oxidation/desorption was used. Equation (1) with the oxidation charge *Q*_Pt_ of HUPD, the scan rate ν during CV, the charge density *ρ* of 2.1 C m_Pt_^−2^, and the mass of platinum *L*_Pt_ served for ECSA calculation.

(1) $$\text{ECSA=}\frac{Q_{\text{Pt}}}{\vartheta \;\cdot \text{ρ }\cdot {\text{ L}}_{\text{Pt}}}$$

The EIS data were further processed by distribution of relaxation times analysis (DRT) using Equation (2), including the ohmic resistance $R_{\infty }$, the polarization resistance $R_{\text{pol}}$, the DRT function *g*, the frequency *f*, and the relaxation time *τ*.^[^ ^18^ ^]^

(2) $${\text{Z(f) =R}}_{\infty }+R_{\text{pol}}\int_{0}^{\infty }\frac{\text{g(τ)}}{\text{1+2iπfτ}}\text{dτ}$$

The DRT function was calculated with DRT tools developed by the Ciucci group and a regularization parameter of 10^−3^. This was chosen to have a trade‐off between low residuals, selectivity, and minimal oscillations for PEMFC. The Gaussian method of discretization was utilized with a first‐order regularization. DRT data were extracted by the use of the Python scipy library and the AIC function from DRT tools to associate impedances with different electrochemical processes. The peak separation and evaluation were carried out by identifying an initial set of peaks, and a vector dataset was created based on these initial peaks. By prioritizing the higher peak heights, a Gaussian fitting was done, and an iterative loop was created through the difference between the fitted data and the output data. The difference was minimized to 10^−6^. The area under the curve was integrated and validated with a composite trapezoidal rule.

### 2.7 Physical Characterization

Thermogravimetric analysis (TGA) of pristine membranes was carried out using TGA 4000 (PerkinElmer) with N_2_ flow rate of 40 mL min^−1^. The temperature program started with 15 min hold at 25 °C, followed by heating from 25 to 100 °C with 10 °C min^−1^, holding at 100 °C for 30 min, and then heating from 100 to 900 °C with 10 °C min ^−1^.

Titration experiments were used to determine the phosphoric acid content of the tested MEAs in comparison with fresh MEAs from the same fabrication batch. MEAs were removed from the cell after finishing HT‐PEMFC operation and immediately prepared for titration experiments by punching out samples with diameters of 8 mm. These samples were measured for thickness and weight. Then, they were put into beaker glasses filled with 70 mL of deionized water and 30 mL of acetone and stirred for 30 min at room temperature. The solution was titrated with 0.1 mol L^−1^ sodium hydroxide using the TA 20 plus device from SI Analytics GmbH (Germany).

Ion chromatography (IC) was used for the analysis of the product water toward phosphate concentration, which was collected during measurements, cooling traps located directly behind the gas outlets, and analyzed after the measurement. The 850 Professional IC from Metrohm AG (Switzerland) was used. A calibration curve with phosphate concentrations of 0.2, 0.75, 1.5, 3, 5, 8 mg L^−1^ was prepared using a standard solution with 1000 mg L^−1^ in water (Fluka). A Metrosep A Supp 5−150/4.0 column and a mixture of 3.6 mmol L^−1^ sodium carbonate and 1.0 mmol L^−1^ sodium hydrogen phosphate were used.

Scanning electron microscopy (SEM) was performed for tested MEAs using JEOL IT800 (Germany) equipped with JEOL energy dispersive X‐ray spectroscopy (EDS), detector. For preparation of MEA cross sections, a small piece of MEA was cut out and assembled between two polystyrene sheets, followed by addition of small amount of acetone on top of the sheets to shrink the sheets and fix the MEA. Then the MEA‐polystyrene composite was broken by hammer blow to get a uniform cross‐section and fixed with carbon tape on a SEM sample holder.

## 3 Results and Discussion

Five membranes were fabricated and compared with the Celtec‐P membrane (Reference), by exploring high‐solid PBI (HS), crosslinked PBI (CL), and inorganic fillers (SiC, Si_3_N_4_, and SiO_2_). The corresponding labeling, as well as the initial membrane thickness after fabrication, and the solid contents, are given in **Table** **1**. The SiC membrane has a comparable thickness to the reference (Celtec P), while the other membrane modifications are 30 to 43 μm thinner. The variation in the thickness results, on the one hand, from manual casting, and on the other hand, from the different viscosities of the solution. However, in an automated process, thicknesses between 250 and 450 μm and a variation of ±20 μm are common. In the case of the HS membrane, the solid content is increased by a factor of 3.7. A further increase in solid content was not explored as the viscosity would be too high to implement this membrane type into the Celtec‐based production process and was therefore less industrially relevant.

**Table 1:** Labeling of tested MEAs based on Celtec technology and membrane thickness after fabrication and solid content.

Membrane and MEA labeling	Specification	Membrane thickness after fabrication/μm	Solid content in membrane/wt%
Reference	Celtec P	360–440	≈5
HS	High solid PBI	317	18.3
CL	Crosslinked PBI	321	7.7
IF SiC	Inorganic filler SiC	360	7.0
IF_Si3N4	Inorganic filler Si_3_N_4_	325	6.4
IF_SiO2	Inorganic filler SiO_2_	330	6.3

For the CL and IF membranes, slightly higher solid contents by a factor of 1.3‐1.5 compared to the reference are found. The filler content was fixed to 2 wt%, as with higher contents, the processing using the Celtec production process, e.g., viscosity and homogeneity, was not given anymore.

Further analysis of thermal stability by TGA of all membranes (see Figure S1 in the Supporting Information) showed stable behavior at 160 °C for all membranes and no impact of modification on the thermal degradation of the PBI, commonly starting above 500 °C.^[^ ^19^ ^]^ For the HS membrane, less water desorption at around 100 °C was observed (≈25 wt% loss) compared to the other membranes (around 40 wt% loss).

**Figure 1:**
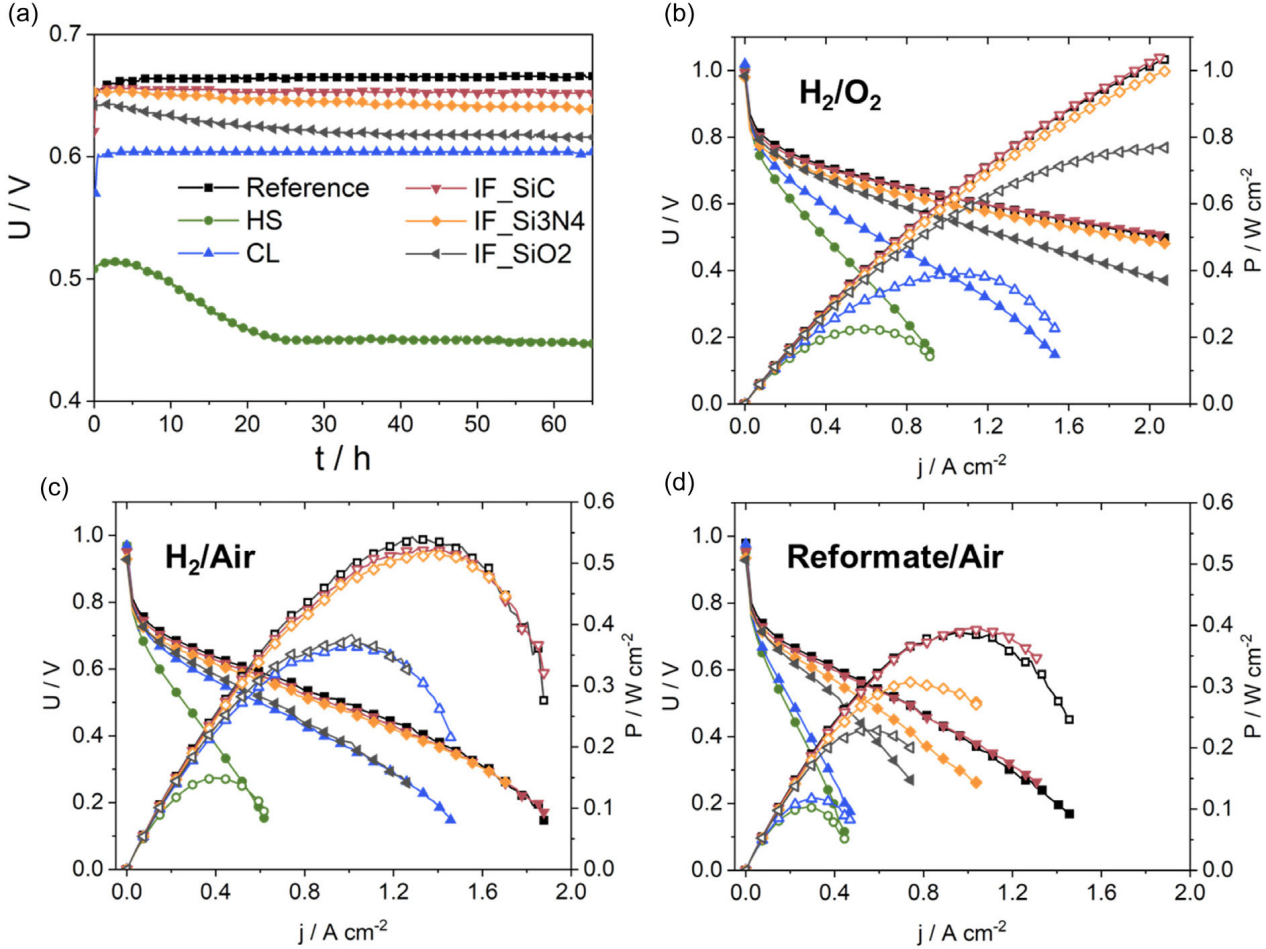
Abbreviations according to Table 1, a) Voltage over time at a constant current density of 0.3 A cm^−2^, and polarization curves with b) H_2_/O_2_, c) H_2_/air, and d) reformate/air operation.

MEAs were fabricated with the modified PBI membranes using similar Celtec‐based assembling and electrodes, followed by HT‐PEMFC operation at 160 °C and ambient pressure. Different behaviors are observed during the break‐in of the MEAs at a constant current density of 0.3 A cm^−2^ in the voltage/time plots in **Figure** **1**a). While the reference and CL have a small increase in voltage by 13 and 11 mV during the first 65 h of operation, the voltage of the other MEAs decreases in the following order: IF_SiC (–4 mV) < IF_Si3N4 (–14 mV) < IF_SiO2 (−26 mV) ≪ HS (−60 mV). A drastic voltage decay of the HS MEA was notably observed within the first 24 h of operation, followed by stabilization. This indicates that irreversible processes occur during the break‐in of this MEA. Possible reasons could be an initial acid redistribution from the membrane to the electrode or acid leaching, which will be checked by titration later (Figure 6a), pin‐hole formation, which would be indicated by low open circuit voltage **Table** **2**), or mechanical collapse. An abrupt mechanical collapse can be excluded by evaluation of the thickness changes of MEAs during break‐in (see Figure S2 in the Supporting Information). A reduction of MEA thickness is monitored over the whole break‐in for all MEAs, independent of modification. No significantly higher thinning is observed for HS MEA. However, for the tested HS MEA polymeric residues after the cell test on the anode flow field are observed (see Figure S3 in the Supporting Information). This was not the case for the other MEAs and indicates chemical instability, e.g., partial dissolution of the HS membrane.

**Table 2:** OCV values and peak power densities determined from polarization curves under different operation gases.

	OCV/V			Peak power density/W cm^−2^		
	H_2_/O_2_	H_2_/air	reformate/air	H_2_/O_2_	H_2_/air	reformate/air
Reference	1.01	0.97	0.98	1.033	0.543	0.388
HS	1.01	0.98	0.95	0.225	0.149	0.103
CL	1.02	0.97	0.98	0.391	0.366	0.117
IF_SiC	0.99	0.95	0.95	1.039	0.523	0.395
IF_Si3N4	0.98	0.93	0.94	0.998	0.521	0.307
IF_SiO2	0.98	0.93	0.93	0.769	0.384	0.228

The performance of the MEAs was first assessed in H_2_/O_2_, H_2_/air, and reformate/air (Figure 1b–d) via polarizations. The open circuit voltage (OCV) was monitored here, as it is affected by hydrogen crossover through the membrane, electronic conduction of the membrane, and the catalyst layer. There, similar values (0.93–1.02 V) are observed for all MEAs under all three gas setups used, indicating no significant hydrogen crossover and excellent membrane integrity (Table 2). Moreover, an impact of the electrodes on OCV is not expected, since similar commercial electrodes were used.

The H_2_/O_2_ performance in terms of peak power density has the following order: IF_SiC ≥ Reference > IF_Si3N4 ≫ IF_SiO2 ≫ CL ≫ HS (Table 2). Slightly higher performance of IF_SiC compared to the reference under H_2_/O_2_ operation was also observed in our previous study.^[^ ^8^ ^]^ Similar trends are observed in H_2_/air, with the exception that the CL and IF_SiO_2_ have comparable performances. While the performance of CL is less affected by the cathode gas, with a decrease of peak power density by only 0.024 W cm^−2^ from O_2_ to air, higher losses of peak power density are found for the reference (–0.490 W cm^−2^), IF_SiC (–0.516 W cm^−2^), IF_Si3N4 (−0.476 W cm^−2^) and IF_SiO2 (−0.386 W cm^−2^). This indicates more severe mass transport issues, which are not visible using oxygen. In the case of CL, fewer mass transport challenges are observed during H_2_/air. However, this might be due to the already low performance and charge transfer limitations under oxygen operation. This could be due to different acid distribution and availability in the CL MEA. The HS MEA still has the lowest performance, which agrees with the drastic voltage drop during the break‐in phase (Figure 1a), indicating mechanical instability (see Figure S3 in the Supporting Information) or irreversible acid redistribution.

The performances were also investigated under reformate/air. Operation with reformate, in general, can induce mass transport limitations at the anode by dilution effect due to the presence of CO and CO_2,_ and mainly affects the anode catalyst in terms of poisoning by CO, but can also cause crossover of CO through the membrane to the cathode.^[^ ^20^ ^]^ While the IF_SiC and the reference show the best performances, IF_Si3N4 has significantly lower performance, followed by IF_SiO_2_. This indicates an impact of the reformate, in terms of anode poisoning and mass transport limitations. This effect might be more pronounced using Si_3_N_4_ and SiO_2_ as inorganic fillers compared to SiC. Furthermore, the performance decay of CL MEA under reformate is significantly higher compared to the other MEAs, so that the performance is now nearly comparable to the low‐performing HS MEA. This indicates stronger problems toward mass transport limitations and catalyst poisoning on the anode, which could be attributed to the lowest acid concentration within MEA, which will be discussed later (Figure 6). A significant CO crossover is excluded for all MEAs as the OCV values of H_2_/air and reformate/air are highly comparable (Table 2).

**Figure 2:**
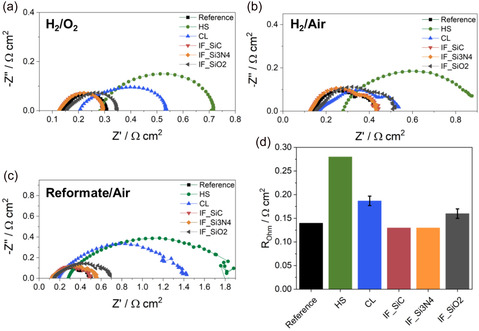
Nyquist plots from EIS at 0.3 A cm^−2^, a) H_2_/O_2_, b) H_2_/air, c) reformate/air, and d) corresponding ohmic resistances *R*_OAhm_ from (a–c) with deviation.

To further elucidate the cause of the performance differences under different fuels and oxidants, EIS data recorded at 0.3 A cm^−2^ in the ohmic‐controlled region were analyzed. First, the ohmic resistances, *R*_Ohm_, were determined from Nyquist plots (**Figure** **2**). *R*_Ohm_ is affected by proton conductivity, membrane, and electronic resistances of electrodes.^[^ ^21^ ^]^ Because the same electrode types were used and the same compression, the differences in *R*_Ohm_ are directly correlated to the ionic conductivity instead of electrical conductivity, which is solely attributed to the differences in membrane properties. The order for *R*_Ohm_ is independent of the operational gases and is as follows (Figure 2d): IF_SiC and IF_Si3N4 < reference < IF_SiO2 < CL < HS. A similar initial membrane thickness of IF_SiC and the reference was observed in Table 1, which does not explain the slightly lower *R*_Ohm_ (difference of 0.01 Ω cm^2^, 7%). In our previous study, we found that a lower *R*_Ohm_ for IF_SiC can be attributed to enhanced proton conductivity by better acid retention of excess phosphoric acid.^[^ ^8^ ^]^ In case of IF_Si3N4, a thinner initial membrane thickness was determined (Table 1), which could result in lower membrane resistance and thus lower *R*_Ohm_. Although IF_SiO2, CL, and HS also have lower initial membrane thicknesses compared to the reference, they display higher *R*_Ohm,_ showing higher membrane resistances. Thus, the incorporation of SiO_2_ and crosslinking leads to slightly higher resistances compared to the reference. For the HS MEA, a 50% higher membrane resistance compared to the reference is observed, which shows the negative impact of the increased solid content and possibly acid leaching, which might have occurred within the first 24 h of operation (Figure 1a), leading to less proton conductivity of the membrane. Moreover, a thicker membrane can also contribute to higher *R*_Ohm_ and was identified by SEM, which will be discussed later and shown in Figure 7b).

**Figure 3:**
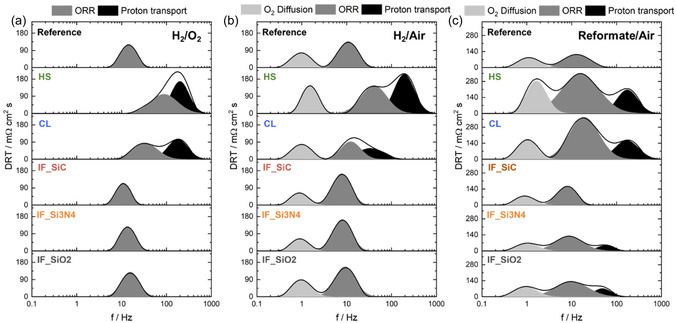
DRT plots from EIS data recorded at 0.3 A cm^−2^ with a) H_2_/O_2_, b) H_2_/air, and c) reformate/air.

The EIS results (Figure 2) were further analyzed using DRT to identify the underlying processes, with peak deconvolution conducted to extract the characteristic frequency and peak area (**Figure** **3**). The peak assignment was done according to Heinzman et al.^[^ ^22^ ^]^ In case of H_2_/O_2_ operation, one main peak attributed to the ORR charge transfer kinetics was identified (100–10 Hz). For the lower performing MEAs, HS, and CL, an additional proton transport peak is also visible, which provides evidence of proton transport limitations on anode and cathode sites, e.g., less acid availability. Similar peaks are found in H_2_/air, with the addition of a low–frequency O_2_ diffusion peak indicating mass transport limitations for all MEAs. This agrees with the decreased performance after switching the cathode gas from O_2_ to synthetic air in Figure 1b,c). During reformate/air operation, next to HS and CL also small proton transport peaks are observed for IF_Si3N4 and IF_SiO2. This points out the first difference of these MEAs to IF_SiC and the reference. To quantify the resistances and rates, the peak area corresponding to the corresponding resistance and the peak position at the center correlating to the rates were determined.

**Figure 4:**
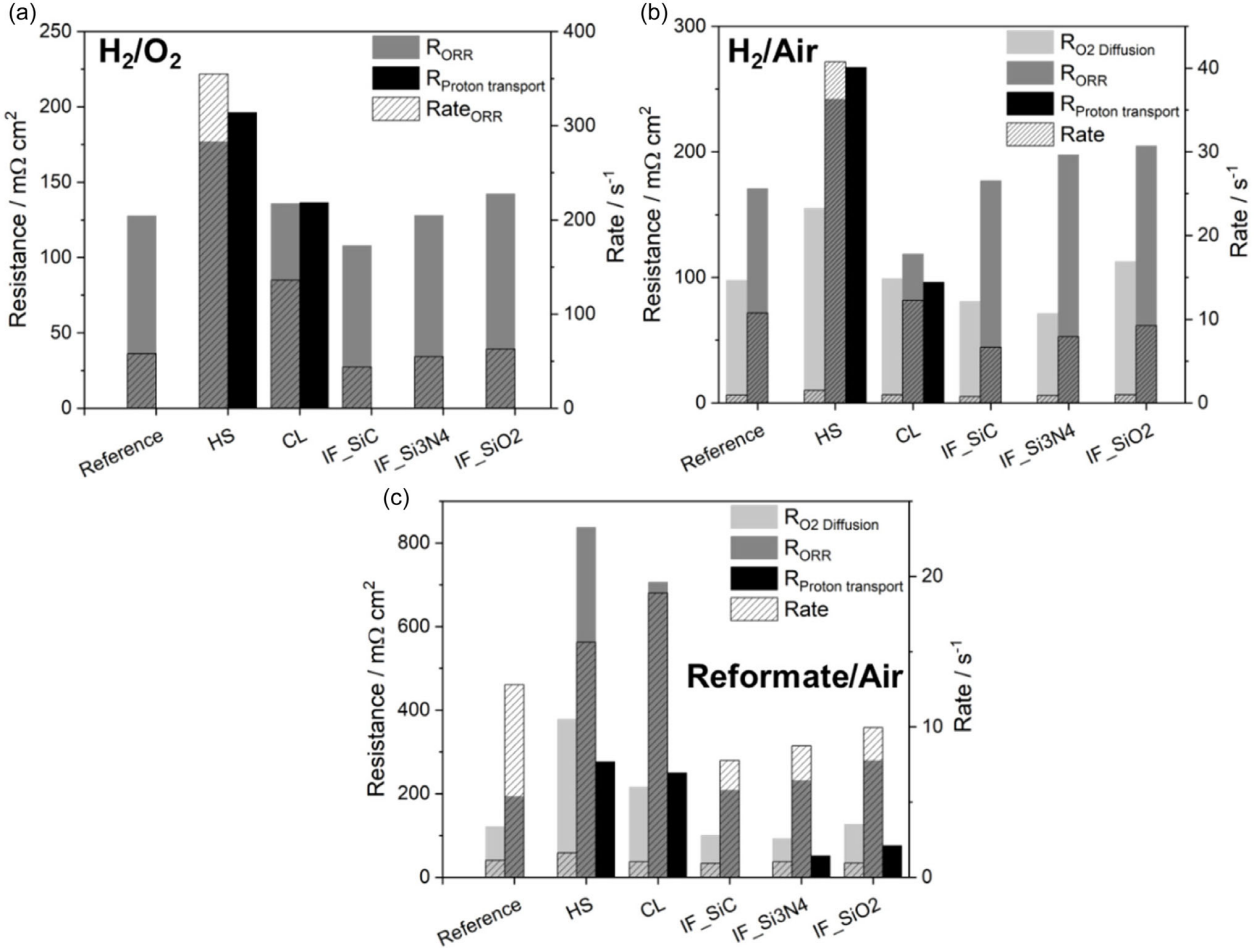
Resistances (peak area) and rates (peak center) determined from DRT plots, a) H_2_/O_2_, b) H_2_/air, and c) reformate/air.

The ORR resistance *R*_ORR_ from the H_2_/O_2_ DRT data (**Figure** **4**a) is the lowest for the highest performing MEA IF_SiC (Figure 1b), followed by IF_Si3N4 < reference < IF_SiO2 < CL ≪ HS. Thus, a direct correlation of *R*_ORR_ with the performance under H_2_/O_2_ is observed. This indicates that the membrane properties impact the ORR kinetics in terms of acid availability and thus proton availability as well as phosphate adsorption at the active catalyst sites,^[^ ^23^, ^24^ ^]^ being best for the IF MEAs and reference. It seems that for CL and HS, fewer protons are available for ORR, which is also consistent with higher proton transport resistance for both MEAs (Figure 3a). With a look at the ORR rates, the highest values are found for the lowest performing MEAs and vice versa. As the ORR peak is related to the kinetics, which follow the Butler–Volmer equation, current density and potential have an impact on the rates, e.g., higher current densities lead to faster kinetics.^[^ ^24^, ^25^ ^]^ Because the EIS data were measured at a fixed current density of 0.3 A cm^−2^, differences in cell voltages were observed. This means that for the low‐performing MEAs, the EIS data are recorded at similar current density but lower electrode potential, and thus, higher overpotentials are present. This explains the higher ORR rates but simultaneously higher resistances.

The DRT results in H_2_/air operation reveal different behaviors compared with H_2_/O_2_. For the diffusion limitations, *R*_O2Diff_ lowest value is found for IF_Si3N4, followed by IF_SiC < CL < reference < IF_SiO2 < HS. Thus, the strong performance decay in the case of the IF MEAs and reference between O_2_ and air operation in Figure 1b,c cannot be solely attributed to mass transport limitations. A similar trend is observed for *R*_ORR,_ with the exception that the CL MEA has the lowest *R*_ORR_. This can explain comparable performances of CL during H_2_/O_2_ and H_2_/air operation in Figure 1b,c. In consequence, the crosslinking leads to an MEA that is less affected by cathode gas in terms of O_2_ diffusion and ORR kinetics, e.g., availability of protons. However, the overall lower performance compared to the reference and IF_SiC and IF_Si3N4 MEAs can be traced back to proton transport resistances, which are still present in contrast to IF and reference MEAs.

For reformate/air, a similar trend to H_2_/air operation is found for *R*_ORR_ and *R*_O2diff_ for reference and IF MEAs, but with higher values for HS and CL, which could possibly be due to effects of the H_2_ diffusion limitations occurring at the anode on the low frequency peak in DRT plot. In case of reformate/air operation next to HS and CL, MEA also a proton transport resistance was also observed for IF_Si3N4 and IF_SiO2. This can be directly referred to the lower performances of both MEAs compared to IF_SiC and the reference when using reformate/air. The proton transport limitations can be attributed to the anode and thus include poisoning of the catalyst, e.g., by CO on the anode, as well as mass transport limitations. However, similar electrodes were used for all MEAs, and the OCV values from Figure 2c do not indicate significant catalyst poisoning and deactivation (values comparable to H_2_/Air operation in Table 2). Therefore, proton availability seems to be lower with reformate operation when Si_3_N_4_ or SiO_2_ was incorporated. CL displayed a drastic performance decay (Figure 1d) using reformate/air, which can be correlated according to the DRT analysis to a higher R_ORR_. So, the reformate penetration also impacts the charge transfer process of this MEA. In summary, DRT enables the deconvolution of different resistances, which explain the performance differences using different operation gases, on the one hand, and different membrane modifications, on the other hand.

**Figure 5:**
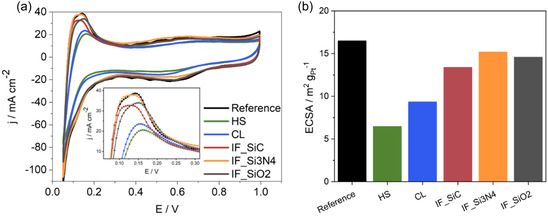
a) Cyclic voltammograms with HUPD region as inset and b) ECSA determined by HUPD.

To further identify the effect of the membrane type on the catalyst accessibility, e.g., wetting and acidifying of the electrode, CVs were recorded for the cathodes. While the IF MEAs and the reference have similar capacitive current densities, the HS and CL MEAs show slightly lower capacitive current densities. This indicates less electrode wetting and agrees with the lower performance and the proton transport resistance observed in DRT analysis for H_2_/air and H_2_/O_2_ operation, in contrast to the other MEAs. Next, the ECSA values were determined via HUPD. For commercial Celtec‐based cathodes, typically, ECSA values in the range of 17‐20 m^2^ g_Pt_^−1^ are reported.^[^ ^17^ ^]^ This agrees with the ECSA of reference MEA shown in **Figure** **5**b of 16.5 m^2^ g_Pt_^−1^. The IF MEAs have slightly lower ECSA values in the range of 15.2 − 13.4 with IF_Si3N4 followed by IF_SiO2 and IF_SiC. For CL and HS, ECSA values below 10 m^2^ g_Pt_^−1^ are observed, indicating less catalyst availability and activity, which is in accordance with the low performance and higher ORR and proton transport resistances. A hindered phosphoric acid distribution could be a reason for that.

**Figure 6:**
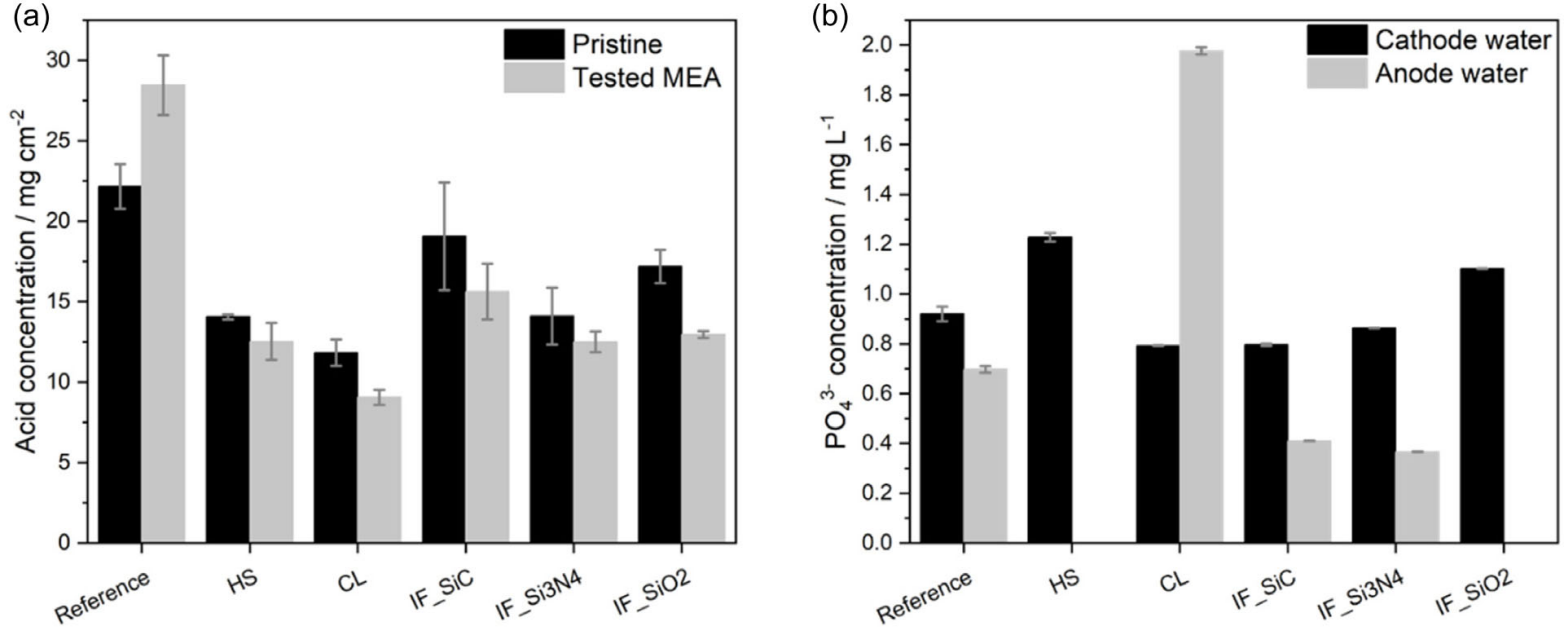
a) Acid concentration of a pristine MEA from the same batch and tested MEAs determined by titration, and b) phosphate concentration in product water of cathode and anode collected at the end of the test determined by IC.

Analysis of the MEAs and their product waters was performed by titration and IC, respectively. The MEA analysis includes the membrane and the electrodes, as these cannot be separated. Comparison of the acid concentration of a pristine MEA from the same batch with the corresponding tested MEA is shown in **Figure** **6**a. For all MEAs except the reference, a lower acid concentration of 11%–25% compared to the pristine MEA from the same batch is found. The reference shows a 28% higher acid concentration compared to a pristine MEA from the same batch, which could be related to inner batch deviation. In general, the acid concentration of the reference is the highest, followed by IF_SiC and IF_SiO2. IF_Si3N4 and HS have similar pristine and tested MEA acid concentrations. However, HS displayed a significantly lower performance and ECSA. Thus, it seems that not the total acid amount but rather the distribution within MEA of HS leads to low performance and high resistance. For example, acid is trapped within the membrane and not distributed through the electrodes, or acid is leached out to the electrodes, leading to less acid in the membrane and thus lower proton conductivity. This would be in accordance with the higher *R*_Ohm_ and the drastic voltage decay observed for HS within the first hours of operation. CL shows the lowest acid concentration, matching the low performance and low ECSA.

IC of the collected product water from the cathode and anode indicated higher phosphate concentrations in the cathode water from HS and IF_SiO2 and the anode water from CL (Figure 6b). For HS, it was only possible to collect water from the cathode due to low performance and lower water production. For IF_SiO2 anode water, no phosphate was detected. The highest phosphate concentration was found at the anode site of CL. The higher concentrations could be an indicator of more pronounced acid leaching. Especially, for CL, this agrees well with the highest acid loss also observed from titration experiments, and the low performance (Figure 6a). For HS acid, leaching does not appear to be the main drive for the lower performance, as already suspected from titration experiments, either the acid is leached only from the membrane to the electrodes, or the membrane undergoes another irreversible process during the first hours of operation which agrees with the observed polymeric residues on the flow fields after test (see Figure S3 in the Supporting Information). For evaluation of dimensional changes of membrane and electrodes, thickness and weight of MEAs before and after FC tests were monitored (Figure S4 and S5 in the Supporting Information). For the initial MEA thickness, the highest value was found for the HS MEA (932 μm), followed by reference (909 μm) and the other MEAs (862–879 μm). As similar electrodes were used, changes in thickness can be mainly related to the membrane. The order of the MEA thickness is similar to the measured membrane thickness after fabrication (Table 1), with exception of HS, which shows a higher thickness after assembling. On the one hand, this could be attributed to inhomogeneities in membrane thickness, and on the other hand, a membrane swelling could have occurred during storage, e.g., between fabrication, MEA assembling, and test. After testing, a reduced thickness by 5% was observed for the reference, while CL and IF MEAs display only a decrease of thickness by 1%–2% indicating stable mechanical behavior. The HS MEA shows an increase of thickness by 1% so that also no mechanical collapse of membrane or thinning is suspected at this point. The measured MEA weights before and after test (Figure S5 in the Supporting Information) display highest values for HS, which agrees with the thickest MEA. After test, weight losses in range of 4%–10% are observed for all MEAs, with exception of HS MEA having a weight gain of 3% which is in accordance with the thickness values after test and the results from product water analysis showing less water production and no acid leaching at the anode.

**Figure 7:**
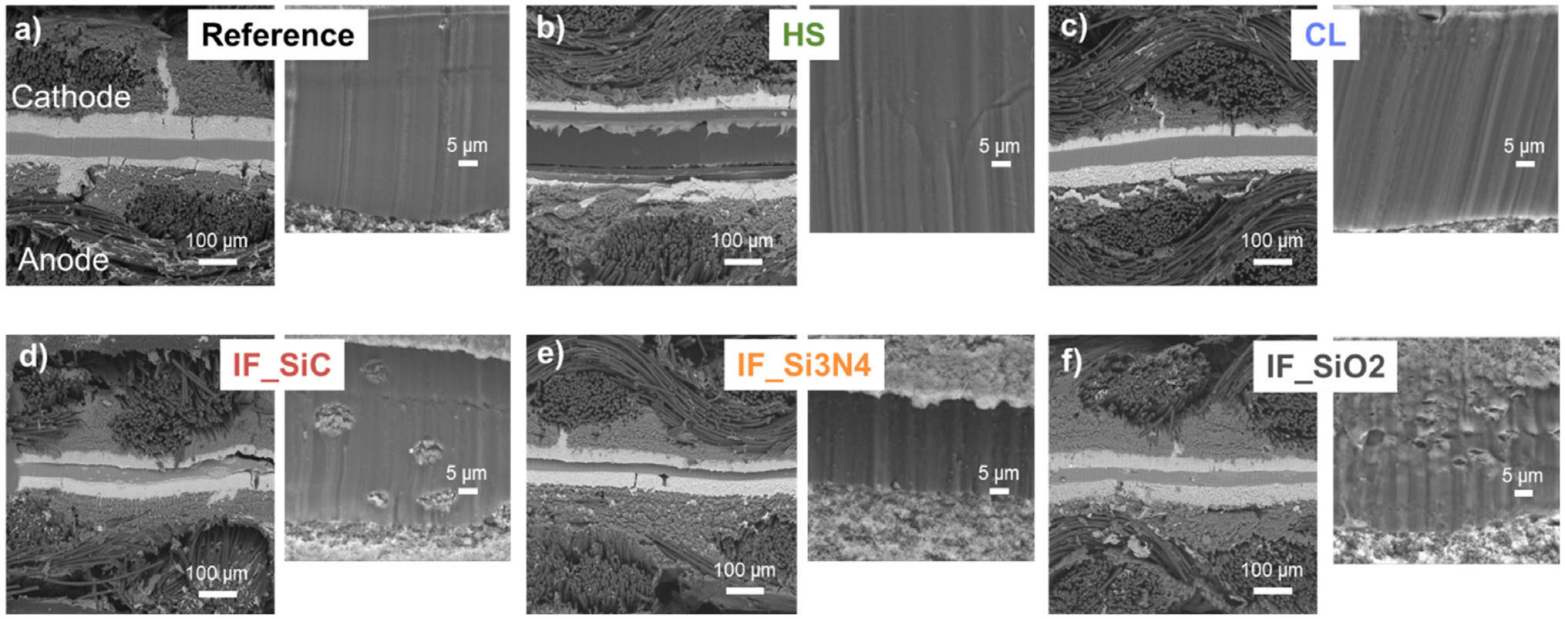
a–f) SEM cross‐sections of MEAs after FC test (cathode up in image) and zoom of membrane.

Last, structural changes were investigated by SEM analysis of the cross‐sections of the tested MEAs (**Figure** **7**). With a look at the membrane‐electrode interface, with exception of the HS MEA, no delamination is observed. For HS MEA, slight delamination of the anode is visible over the whole cross‐section. This is a hint for low electrode‐membrane interaction and could be reason for the poor performance of this MEA. Also, it is visible from SEM images that the HS membrane is thicker compared to the other membranes, which contributes to higher ohmic resistance (Figure 2).

While for reference, HS and CL membranes have no particles visible and expected (Figure 7a–c), for the IF membranes, particles and particle agglomerates within the membrane can be seen (Figure 7d–f). These particles were identified as the silica‐based fillers by EDS (Figure S6 in the Supporting Information). Thus, it can be concluded that the IF membranes and fillers are stable under HT‐PEMFC conditions within this operation of around 80 h.

## 4 Conclusions

Five membranes based on the commercial Celtec‐P technology were modified to increase the solid content while maintaining the industrially relevant production process. This included insertion of inorganic fillers and crosslinking for an increase of solid content by ≈2 wt% and a high‐solid content membrane with an increase of 13 wt%. Identical MEA fabrication for modified and standard Celtec MEAs was performed, followed by comparison of HT–PEMFC performance. The comparison to commercial reference shows:

*SiC filler:* A comparable performance to commercial Celtec MEA was reached. Better membrane resistance was identified.

*Si_3_N_4_ filler:* Slightly lower performance was found when operating with H_2_/O_2_ and H_2_/air, but low performance was observed when operating with reformate/air. This was attributed to proton transport limitations.

*SiO_2_ filler:* Lower performance compared to SiC and Si_3_N_4_, especially under reformate/air operation. This was attributed to proton transport and mass transport limitations, possibly due to the hygroscopic character of SiO_2_ and gas penetration to the membrane.

*Crosslinking:* Low performance and ECSA due to proton transport limitations, but less affected by cathode gas variation between oxygen and air. Not suitable for reformate operation due to strong mass transport limitations.

*High solid content:* Not stable in HT‐PEMFC conditions. Voltage drop within the first day of operation and low performance, and ECSA. Acid leaching from the membrane to the electrodes and an irreversible process leading to delamination of MEA is assumed, resulting in higher membrane resistance.

In summary, SiC as an inorganic filler was the best candidate for membrane modification. It was also shown that this membrane is more stable during a 1000 h load‐cycling test compared to standard Celtec MEA.^[^ ^8^ ^]^ However, the positive impact of crosslinking on the cathode gas tolerance could be further focused and optimized, possibly in combination with SiC filler. A systematic investigation of the degree of crosslinking, as well as the amount of filler and the particle size, would be helpful in future. However, the applicability of CL approach on a large scale has to be considered due to the use of hazardous glutaraldehyde and the need for a continuous oven process. Furthermore, the HS membrane approach could be optimized toward slightly lower solid content to check possible performance improvements. Moreover, for all membranes, tolerance toward reformate could be improved. The possibilities for integration of different membrane modifications in industrial fabrication processes shown in this study can be adapted for other novel membranes to enhance the comparability between different studies and accelerate the upscaling process.

## Conflict of Interest

The authors declare no conflict of interest.

## Supporting information

Supplementary Material

## Data Availability

The data that support the findings of this study are available from the corresponding author upon reasonable request.
