# Supplementary material for: Performance of High Temperature Polymer Electrolyte Membrane Fuel Cells as a Function of Polybenzimidazole Membrane Modification
Source: ChemSusChem. 2025 Oct 21;18(24):e202501575. doi: 10.1002/cssc.202501575 (PMC12703420; doi:10.1002/cssc.202501575)
Supplement: Supplementary file 1 — Supplementary Material [file CSSC-18-e202501575-s001.pdf]

Supporting Information

Performance of High Temperature PEM Fuel Cells as a Function of PBI Membrane Modification

Julia Müller-Hülstede[a*], Dana Schonvogel[a], Julian Büsselmann[a], Jörg Belack[b], Jurica Vidakovic[c], Md Raziun B. Mamtaz[d], Quentin Meyer[d], Chuan Zhao[d], and Peter Wagner[a]

[a] German Aerospace Center (DLR), Institute of Engineering Thermodynamics

Carl-von-Ossietzky-Str. 15, 26129 Oldenburg, Germany

[b] BASF Catalysts Germany GmbH, Pettenkoferstrasse 9, 67063 Ludwigshafen am Rhein, Germany

[c] Trigona Fuel Cell Components GmbH, Kasteler Straße 45, 65203 Wiesbaden, Germany

[d] University of New South Wales, School of Chemistry, NSW, 2052, Sydney, Australia

Corresponding author:

Julia.mueller-huelstede@dlr.de

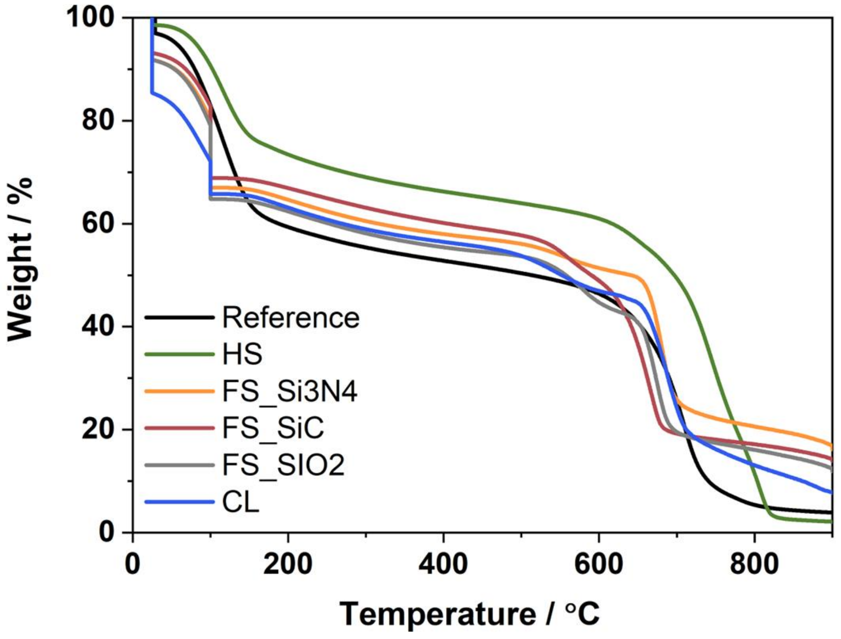

Figure 1: Thermogravimetric analysis of pristine membranes. Program: 15 min hold at 25 °C, 25-100 °C with 10 °C min^{-1}, 30 min at 100 °C, 100-900 °C with 10 °C min^{-1} with N_2 flow rate of 40 mL min^{-1}.

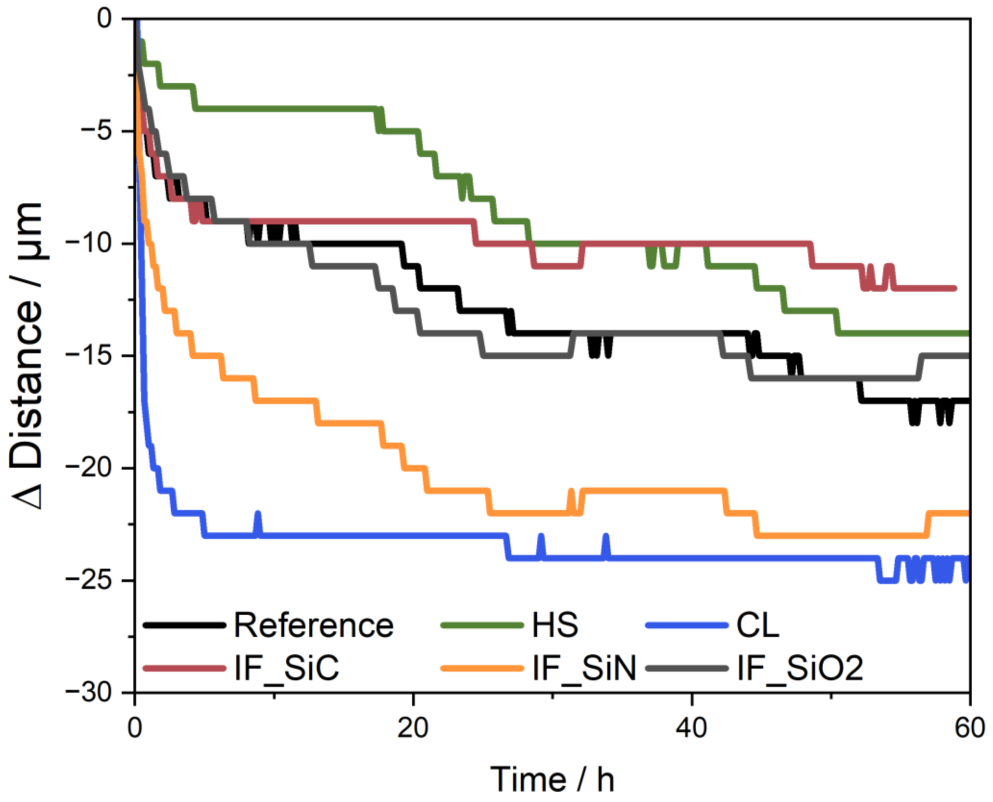

Figure 2: Distance changes of MEA during the break-in procedure at constant current density of 0.3 A cm^{-2} under H2/air (1.5/2.0) monitored using CMD V1.1 unit from balticFuelCells.

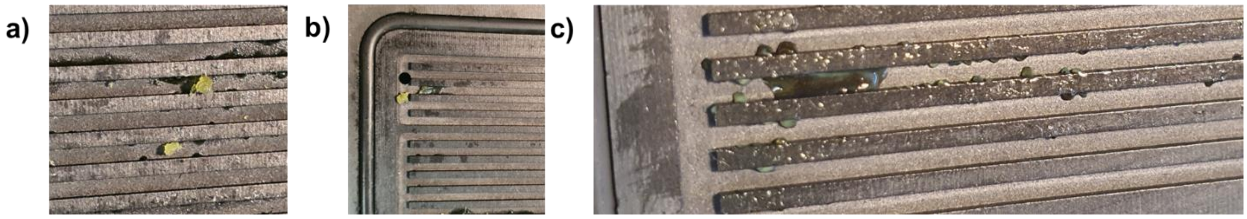

Figure 3: a) -c) Residues observed on the anode flow fields after independent test of two HS MEAs from the same batch.

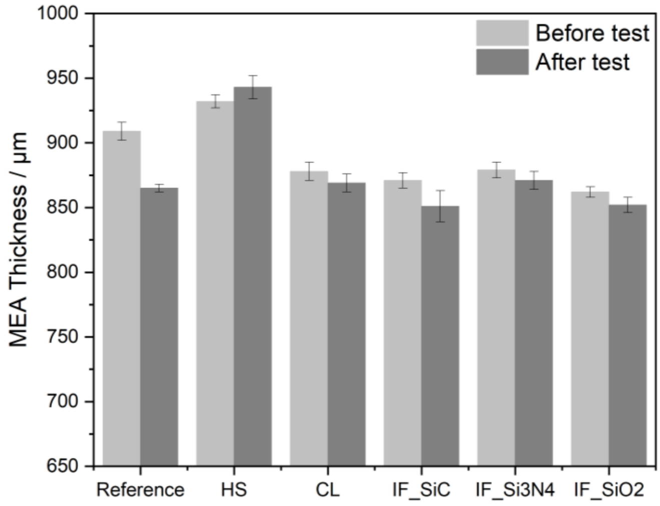

Figure 4: Thickness measured for each MEA before and after test with standard deviation of at least three values.

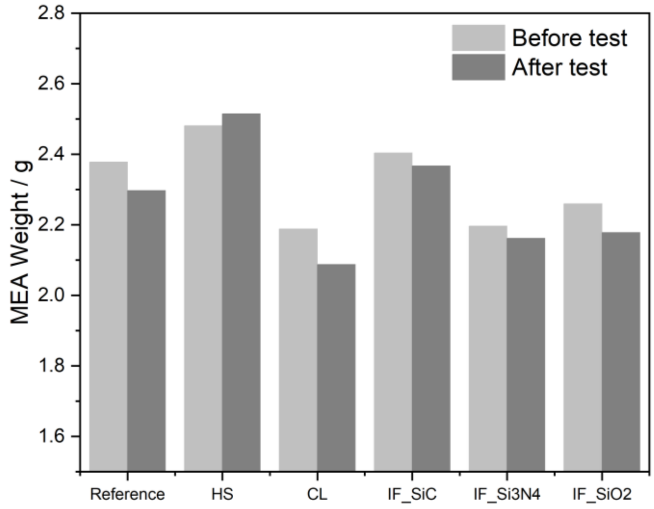

Figure 5: Weight of MEAs before and after test.

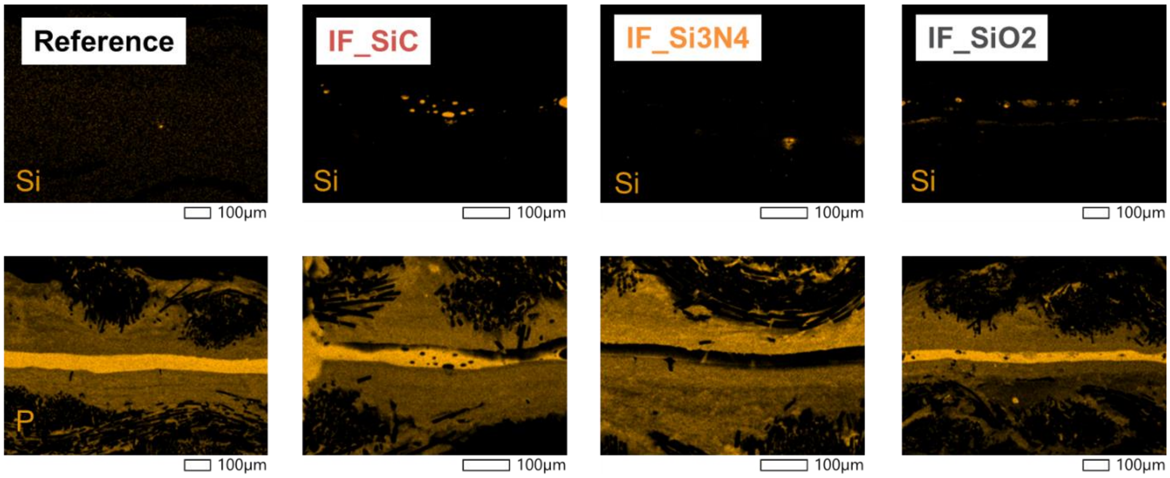

Figure 6: EDS mapping of Si and P for reference and IF MEAs.
